# Four *Sordariomycetes* freshwater hyphomycetes from the Chishui River Basin in Guizhou Province, China

**DOI:** 10.3897/mycokeys.131.185643

**Published:** 2026-04-29

**Authors:** Hong Zhang, Ya-Ru Sun, Yongjian Luo, Jiayi Zhang, Tianyuan Fan, Xudong Liu, Ning-Guo Liu, Yu Mu

**Affiliations:** 1 School of Food Engineering, Moutai Institute, Renhuai, 564507, China School of Food and Pharmaceutical Engineering, Guizhou Institute of Technology Guiyang China; 2 Department of Light Industry and Chemical Engineering, Guizhou Light Industry Polytechnic University, Guiyang 550025, China School of Food Engineering, Moutai Institute Renhuai China; 3 Guizhou Engineering Research Center for Health-Functional Liquor Brewing Technology, Moutai Institute, Renhuai, 564507, China Department of Light Industry and Chemical Engineering, Guizhou Light Industry Polytechnic University Guiyang China; 4 School of Food and Pharmaceutical Engineering, Guizhou Institute of Technology, Guiyang 550025, China Guizhou Engineering Research Center for Health-Functional Liquor Brewing Technology, Moutai Institute Renhuai China

**Keywords:** *
Chaetosphaeriaceae
*, freshwater fungi, phylogeny, *
Rhamphoriaceae
*, taxonomy

## Abstract

During a survey investigating fungal diversity in the Chishui River Basin, Guizhou Province, China, four fungal specimens were collected from submerged wood in freshwater habitats. Based on detailed morphological observations and multi-gene phylogenetic analyses, two novel species, *Chloridium chishuiensis* and *Xylolentia chishuiensis*, are herein described and illustrated in detail. *Chloridium chishuiensis* is phylogenetically closely related to *C. crousii*, *C. kirkii*, *C. tropicale*, and *C. xishuangbannaense* but differs from these taxa by producing multiple conidiogenous loci and possessing longer conidiophores. *Xylolentia chishuiensis* forms a sister lineage to *X. oblongispora* and is distinguished by its smaller, ellipsoid to subglobose conidia. Additionally, a new host record of *Rhamphoriopsis aquimicrospora* is reported, accompanied by a new geographical record of *Xylolentia yibinensis*. These findings enriched the fungal diversity of the Chishui River Basin.

## Introduction

Freshwater fungi represent a diverse and ecologically significant group that colonize a wide range of aquatic environments, including rivers, lakes, ponds, and streams (Li et al. 2017). They play essential roles in nutrient cycling and serve as a vital food source for invertebrates and other aquatic organisms (Covich et al. 1999). In addition, their sensitivity to environmental changes makes them valuable bioindicators for assessing water quality and pollution levels (Luo et al. 2019; Shen et al. 2025). As a polyphyletic assemblage, freshwater fungi encompass members of *Ascomycota*, *Basidiomycota*, and *Chytridiomycota* (Calabon et al. 2022; Bao et al. 2023; Shen et al. 2025). In Guizhou Province, an increasing number of freshwater fungal taxa have been documented in recent years, reflecting growing research interest in this region (Yang et al. 2023; Liu et al. 2024; Bao et al. 2025; Xiao et al. 2025).

*Chloridium* was established by Link (1809) with *C. viride* as the type species. The genus is characterized by brown, unbranched conidiophores, phialidic conidiogenous cells with funnel-shaped collarettes, and aseptate, hyaline to pale brown conidia (Seifert et al. 2011; Réblová et al. 2022; Lin et al. 2025). Species of *Chloridium* are widely distributed in terrestrial and freshwater habitats worldwide, where they occur as saprobes on decaying plant material (Luo et al. 2019; Wu and Diao 2022; Liu et al. 2025; Shen et al. 2025). In China, reports of this genus have been predominantly from freshwater environments in the southwestern region (Liu et al. 2025; Shen et al. 2025).

*Rhamphoriopsis* was introduced by Réblová and Štěpánek (2018), based on the type species *R. muriformis*, isolated from decaying wood of *Buxus sempervivens* in France. Subsequently, several additional *Rhamphoriopsis* species have been described as saprobes, with most records originating from Guizhou and Yunnan Provinces, China (Lin et al. 2025; Liu et al. 2025). Currently, the genus accommodates twelve species, the majority of which are defined based on their hyphomycetous asexual morphs, featuring macronematous, mononematous, or sometimes synnematous conidiophores, polyblastic, integrated, terminal, cylindrical conidiogenous cells with numerous denticles, and ellipsoidal to obovoid, hyaline, aseptate conidia (Réblová and Štěpánek 2018; Hyde et al. 2020; Lin et al. 2025; Liu et al. 2025; Index Fungorum 2026). A few species are known by their sexual morphs, which are characterized by perithecial, nonstromatic, immersed ascomata with cylindrical necks, unitunicate, 8-spored asci with a distinct non-amyloid apical ring, and ellipsoidal to fusiform, smooth-walled ascospores (Réblová and Štěpánek 2018).

*Xylolentia* was described by Réblová and Štěpánek (2018), with *X. brunneola* as the type species. The asexual morph of *Xylolentia* is characterized by macronematous, mononematous, unbranched, septate conidiophores, polyblastic conidiogenous cells with a sympodially extending rachis, and ellipsoidal to obovoid, aseptate conidia that are hyaline when young, turning brown when mature (Réblová and Štěpánek 2018; Yuan et al. 2020; Liu et al. 2025; Shen et al. 2025; Xu et al. 2025). The sexual morph features globose, glabrous, black ascomata with a cylindrical neck; unitunicate, cylindric-clavate, long pedicellate asci with a distinct, inamyloid apical ring; and ellipsoidal to obovoid, brown, septate ascospores (Réblová and Štěpánek 2018). *Xylolentia* is distinguished from other genera in *Rhamphoriaceae* by its production of uni-septate, brown ascospores (Réblová and Štěpánek 2018). Recently, ten new *Xylolentia* species have been described, primarily from China (Guangdong, Guizhou, Sichuan, and Yunnan Provinces and the Tibet Autonomous Region), where they occur as saprobes on various decaying plant substrates in both freshwater and terrestrial habitats (Wu and Diao 2022; Kirschner and Hsieh 2023; Lin et al. 2025; Liu et al. 2025; Zhao et al. 2025).

In this study, four hyphomycetous taxa are described using integrated morphological and phylogenetic approaches. Two new species in the genera *Chloridium* and *Xylolentia* are introduced, a new host record for *Rhamphoriopsis aquimicrospora* is reported, and a new geographical record for *Xylolentia yibinensis* is provided. This work expands the known fungal diversity of the Chishui River Basin and provides foundational data for future research on microbial resources in this ecologically distinctive region.

## Materials and methods

### Specimen collection, examination, and isolation

Samples of submerged decaying wood were collected in the Chishui River (27.5°N, 106.2°E), Guizhou Province, China. The samples were packaged in plastic bags and brought to the laboratory. The sporocarps on natural substrates were observed and photographed using a stereomicroscope (SteREO Discovery V12, Carl Zeiss Microscopy GmbH, Germany). Micro-morphological characteristics were observed and photographed using a Nikon ECLIPSE Ni compound microscope (Nikon, Japan) equipped with a Nikon DS-Ri2 digital camera (Nikon, Japan). Adobe Photoshop CS6 Extended v. 13.0 software was used to make photo plates. Measurements were performed with Tarosoft Image Frame Work software.

Single-spore isolations were used to obtain pure cultures. Germinated conidia were transferred to new potato dextrose agar (PDA) plates and incubated at room temperature for four weeks (Senanayake et al. 2020). The pure cultures obtained were deposited in the Guizhou Culture Collection (**GZCC**), Guiyang, China. Herbarium materials were deposited in the Herbarium of Cryptogams, Kunming Institute of Botany, Academia Sinica (**KUN-HKAS**), Kunming, China, and the Herbarium of Guizhou Academy of Agricultural Sciences (**GZAAS**), Guiyang, China. The scientific names of the new species were registered in Fungal Names (https://nmdc.cn/fungalnames/registe; accessed on 10 April 2026).

### DNA extraction, PCR amplification, and sequencing

Fresh fungal mycelia grown on PDA medium were scraped with a sterile scalpel. Genomic DNA was extracted from scraped mycelia using the BIOMIGA Fungus Genomic DNA Extraction Kit (GD2416, BIOMIGA, San Diego, CA, USA), following the manufacturer’s protocol. Five gene regions were selected for PCR amplification in this study: the nuclear ribosomal 28S large subunit (LSU), the internal transcribed spacer (ITS: ITS1–5.8S–ITS2), the nuclear ribosomal 18S small subunit (SSU), the RNA polymerase second largest subunit (*rpb*2), and the translation elongation factor 1-alpha (*tef*1-α). Polymerase chain reaction (PCR) was performed in a total volume of 20 µL, containing 10 µL of 2× PCR Master Mix, 7 µL of ddH_2_O, 1 µL of each primer, and 1 µL of template DNA. The primers and specific thermocycling conditions for each gene region are detailed in Table 1. Following purification of the PCR products using a high-affinity purification (HAP) method, Sanger sequencing was performed by SinoGenoMax Co., Ltd. (Beijing, China).

**Table 1.** Primers and PCR procedures used in this study.

Locus	Primers		PCR procedures	Reference
	Name	Sequence (5’-3’)		
Nuclear ribosomal 28S large subunit (LSU)	LR0R	ACCCGCTGAACTTAAGC	94 °C–3 min; 94 °C–30 s; 52 °C–30 s; 72 °C–1 min; Repeat 2–4 for 35 cycles; 72 °C–8 min; 4 °C on hold	Vilgalys and Hester (1990), Rehner and Samuels (1994)
	LR5	TCCTGAGGGAAACTTCG		
Nuclear ribosomal 18S small subunit (SSU)	NS1	GTAGTCATATGCTTGTCTC		White et al. (1990)
	NS4	CTTCCGTCAATTCCTTTAAG		
Internal transcribed spacer (ITS: ITS1-5.8S-ITS2)	ITS5	GGAAGTAAAAGTCGTAACAAGG		
	ITS4	TCCTCCGCTTATTGATATGC		
RNA polymerase second largest subunit (*rpb*2)	fRPB2-5F	GAYGAYMGWGATCAYTTYGG	94 °C–3 min; 94 °C–20 sec; 55 °C–30 sec; 72 °C–1 min; Repeat 2–4 for 40 cycles; 72 °C–10 min; 4 °C on hold	Liu et al. (1999)
	fRPB2-7cR	CCCATRGCTTGYTTRCCCAT		
The translation elongation factor-1 alpha (*tef*1-α)	EF1-983F	GCYCCYGGHCAYCGTGAYTTYAT	94 °C–2 min; 66 °C–56 °C (touchdown 9 cycles); 94 °C–30 sec; 56 °C–1 min; 72 °C–1 min; Repeat 3–5 for 36 cycles; 72 °C–10 min; 4 °C on hold	Rehner and Buckley (2005)
	EF1-2218R	ATGACACCRACRGCRACRGTYTG		

### Phylogenetic analyses

BLASTn (https://blast.ncbi.nlm.nih.gov//Blast.cgi) was used to evaluate closely related strains to the new taxa. Related sequences were downloaded from GenBank according to published literature (Wu and Diao 2022; Liu et al. 2025; Shen et al. 2025; Zhao et al. 2025) (Table 2). Multiple alignments were automatically performed using online MAFFT version 7 (https://mafft.cbrc.jp/alignment/server/index.html) (Katoh and Standley 2019). Trimal v1.2 (Capella-Gutiérrez et al. 2009) was used to remove ambiguously aligned regions and uninformative positions with the gappyout option. Five gene regions were combined using SequenceMatrix 1.7.8 (Vaidya et al. 2011). Alignments were checked visually using AliView (Larsson 2014). Sequences derived in this study were deposited in GenBank.

**Table 2.** Taxa used in the phylogenetic analysis with the corresponding GenBank accession numbers. The newly generated strains are indicated in bold. N/A: Not available. T: Ex-type strain.

Species	Strain/Isolate	Type	ITS	LSU	SSU	*tef*1-α	*rpb*2
*Chloridium bellum* var. bellum	CBS 709.73A	T	OP455360	OP455466	NA	OP464934	NA
*Chloridium bellum* var. bellum	CBS 709.73B		OP455361	OP455467	NA	OP464935	NA
*Chloridium bellum* var. *luteum*	CBS 141.54	T	OP455362	OP455469	NA	OP464936	NA
*Chloridium biforme*	ICMP 23429	T	OP455363	OP455470	NA	OP464937	NA
*Chloridium caesium*	CBS 230.74		OP455364	OP455471	NA	OP464938	NA
*Chloridium caesium*	CBS 102339		OP455365	OP455472	NA	OP464939	NA
*Chloridium caudigerum*	CBS 248.75		OP455368	OP455475	NA	OP464942	NA
*Chloridium caudigerum*	CBS 263.76B		OP455369	OP455476	NA	OP464943	NA
***Chloridium chishuiensis***	**GZCC 26-0008**	**T**	**PX915484**	**PZ162726**	**NA**	**NA**	**NA**
*Chloridium chlamydosporum*	CBS 114.41	T	OP455385	OP455492	NA	OP464958	NA
*Chloridium chlamydosporum*	CBS 239.75C		OP455386	OP455493	NA	OP464959	NA
*Chloridium chloridioides* var. chloridioides	CBS 129.72		OP455393	OP455500	NA	OP464966	NA
*Chloridium chloridioides* var. chloridioides	CBS 239.75A		OP455394	OP455501	NA	OP464967	NA
*Chloridium chloridioides* var. *convolutum*	CBS 145504	T	OP455395	OP455502	NA	OP464969	NA
*Chloridium chloridioides* var. *convolutum*	CBS 145636		OP455396	OP455503	NA	OP464970	NA
*Chloridium chloroconium*	CBS 149055		OP455398	OP455505	NA	OP464972	NA
*Chloridium costaricense*	CBS 409.94	T	OP455399	OP455506	NA	OP464973	NA
*Chloridium crousii*	CGMCC 3.20701	T	OL627543	OL655003	NA	NA	NA
*Chloridium culmicola*	CGMCC 3.20639	T	OL627650	OL655054	NA	NA	NA
*Chloridium cuneatum*	GZCC 20-0005	T	MN999924	MN901120	NA	NA	NA
*Chloridium cylindrosporellum*	CGMCC 3.20719	T	OL627899	NA	NA	NA	NA
*Chloridium detriticola* var. detriticola	CBS 345.67	T	MH858992	MH870689	NA	OP464974	NA
*Chloridium detriticola* var. detriticola	CBS 581.73		OP455400	OP455507	NA	OP464975	NA
*Chloridium detriticola* var. *effusum*	ICMP 15144	T	OP455402	OP455509	NA	OP464977	NA
*Chloridium elongatum*	CBS 147816	T	OP455403	OP455510	NA	OP464978	NA
*Chloridium fuscum*	CBS 169.27	T	OP455404	OP455511	NA	OP464979	NA
*Chloridium fuscum*	CBS 195.60		OP455405	OP455512	NA	OP464980	NA
*Chloridium gamsii*	CBS 667.75	T	OP455415	OP455522	NA	OP464990	NA
*Chloridium guttiferum*	CBS 126073	T	MH864068	MH875524	NA	OP464991	NA
*Chloridium humicola*	CBS 218.86		OP455416	OP455523	NA	OP464992	NA
*Chloridium humicola*	CBS 420.73	T	OP455417	OP455524	NA	OP464993	NA
*Chloridium hydei*	KUNCC 23-13787A	T	PQ845788	PV536240	NA	PX238200	NA
*Chloridium jilinense*	CGMCC 3.20640	T	OL627659	OL655058	NA	NA	NA
*Chloridium kirkii*	CGMCC 3.20703	T	OL627588	OL655020	NA	NA	NA
*Chloridium mirabile*	CBS 408.76	T	OP455420	OP455526	NA	OP464994	NA
*Chloridium mirabile*	CBS 149309		OP455419	OP455527	NA	OP464995	NA
*Chloridium moratum*	CBS 127627	T	OP455421	OP455528	NA	OP464996	NA
*Chloridium moratum*	FMR 11343		OP455422	OP455529	NA	OP464997	NA
*Chloridium narathiwatense*	MFLUCC 24-0595	T	PV271894	PV271933	NA	NA	NA
*Chloridium novae-zelandiae*	ICMP 22736	T	OP455423	OP455530	NA	OP464998	NA
*Chloridium peruense*	CBS 126074	T	OP455424	OP455531	NA	OP464999	NA
*Chloridium setosum*	CGMCC 3.20741	T	OL628345	NA	NA	NA	NA
*Chloridium setosum*	CBS 263.76A		OP455427	OP455534	NA	OP465002	NA
*Chloridium shangsiense*	CGMCC 3.20632	T	OL627574	OL655016	NA	NA	NA
*Chloridium sinense*	CGMCC 3.20743	T	OL628375	NA	NA	NA	NA
*Chloridium* sp.	CBS 148972		OP455453	OP455561	NA	OP465025	NA
*Chloridium* sp.	CBS 148973		OP455454	OP455562	NA	OP465026	NA
*Chloridium subglobosum*	CBS 134152		OP455425	OP455532	NA	OP465000	NA
*Chloridium subglobosum*	CBS 696.74	T	OP455426	OP455533	NA	OP465001	NA
*Chloridium tongrense*	GMB5335		PV932957	PV932976	NA	NA	NA
*Chloridium tropicale*	CGMCC 3.20725	T	OL628018	OL655153	NA	NA	NA
*Chloridium virescens*	CBS 424.76		OP455428	OP455535	NA	OP465003	NA
*Chloridium virescens*	CBS 601.75B		OP455429	OP455536	NA	OP465004	NA
*Chloridium volubile*	CBS 144661	T	OP455446	OP455554	NA	OP465018	NA
*Chloridium volubile*	ICMP 22553		OP455447	OP455555	NA	OP465019	NA
*Chloridium xishuangbannaense*	CGMCC 3.20723	T	OL628006	OL655149	NA	NA	NA
*Chloridium ypsilosporum*	CBS 121859		OP455452	OP455560	NA	OP465024	NA
*Chloridium yunnanense*	KUNCC 23-13474	T	PQ845789	PV536241	NA	PX238201	NA
*Menispora caesia*	CBS 144659		MW984578	MW984560	NA	OL654038	NA
*Menispora tortuosa*	CBS 117553		OL654111	OL654169	NA	OL654044	NA
*Myrmecridium schulzeri*	CBS 100.54		EU041769	EU041826	NA	NA	NA
*Myrmecridium sorbicola*	CBS 143433		MH107901	MH107948	NA	NA	NA
*Rhamphoria pyriformis*	CBS 139033		KT991677	KT991665	MG600406	NA	KT991656
*Rhamphoria pyriformis*	CBS 139024	T	MG600392	MG600397	MG600405	NA	MG600401
*Rhamphoriopsis aquimicrospora*	GZCC 20-0515	T	OP377812	OP377911	OP377996	OP472992	OP473085
*Rhamphoriopsis aquimicrospora*	KUNCC 24-18174		PV932469	PV932447	NA	NA	NA
*Rhamphoriopsis aquimicrospora*	GZCC 18-0055		PQ898766	PQ898802	PQ898833	PV040816	NA
*Rhamphoriopsis aquimicrospora*	HKAS 146950		PV583404	NA	NA	NA	NA
*Rhamphoriopsis aquimicrospora*	N3		PX927613	PZ000583	NA	NA	NA
***Rhamphoriopsis aquimicrospora***	**GZCC 26-0014**		**PX927612**	**PZ000586**	**PZ071918**	**NA**	**NA**
*Rhamphoriopsis glauca*	CBS 480.75		NA	MH872702	NA	NA	NA
*Rhamphoriopsis hyalospora*	MFLU 19-2849	T	MN846344	MN846342	NA	NA	NA
*Rhamphoriopsis muriformis*	CBS 131269	T	NA	MG600396	MG600404	NA	MG600400
*Rhamphoriopsis muriformis*	CBS 127683		MG600389	MG600395	MG600403	NA	MG600399
*Rhamphoriopsis sympodialis*	GZCC 18-0095		MT079187	MT079191	NA	NA	NA
*Rhamphoriopsis synnematosa*	CPC 45231		OR680773	OR717029	NA	NA	OR683730
*Rhodoveronaea aquatica*	MFLUCC 18-1339	T	MK828641	MK849785	MK828310	MN194046	NA
*Rhodoveronaea aquatica*	GZCC 20-0447		OP377862	OP377947	OP378027	OP473041	OP473107
*Rhodoveronaea everniae*	CBS 148309	T	OK664737	OK663776	NA	NA	OK651172
*Rhodoveronaea hainanensis*	GZCC 22-2020	T	OP748935	OP748932	NA	NA	NA
*Rhodoveronaea hyalina*	GZCC 23-0622	T	PP102206	PP102207	PP102214	PP259403	PP259399
*Rhodoveronaea hyalina*	GZCC 23-0623		PP102211	PP102208	PP102215	PP259404	PP259400
*Rhodoveronaea lignicola*	GZCC 23-0624	T	PP102212	PP102209	PP102216	PP259405	PP259401
*Rhodoveronaea lignicola*	GZCC 23-0625		PP102213	PP102210	PP102217	PP259406	PP259402
*Rhodoveronaea nieuwwulvenica*	CBS 149447	T	OQ628466	OQ629048	NA	OQ627955	OQ627935
*Rhodoveronaea varioseptata*	CBS 431.88	T	EU041813	EU041870	NA	NA	NA
*Rhodoveronaea varioseptata*	CBS 123472		MG600393	FJ617559	MG600408	NA	JX066701
*Rhodoveronaea varioseptata*	CBS 123473		KT991676	FJ617560	JX066710	NA	JX066700
*Xylolentia bambusae*	ZHKUCC 24-1142	T	PQ376583	PQ376584	PQ380133	PQ383292	PQ383291
*Xylolentia brunneola*	PRA-13611	T	MG600394	MG600398	MG600407	NA	MG600402
***Xylolentia chishuiensis***	**GZCC 26-0001**	**T**	**PX927610**	**PZ000584**	**PZ071916**	**NA**	**NA**
*Xylolentia hydei*	KUNCC 23-13819	T	PQ845758	PV536235	PX218386	PX238324	PX233777
*Xylolentia matsushimae*	NN043170		OL627569	NA	NA	NA	NA
*Xylolentia aseptata*	GZCC 20-0424	T	OP377859	OP377944	OP378024	OP473038	OP473104
*Xylolentia aseptata*	GZCC 20-0426		OP377860	OP377945	OP378025	OP473039	OP473105
*Xylolentia oblongispora*	GZCC 18-0054	T	PQ898745	PQ898781	PQ898815	PV040797	NA
*Xylolentia palmicola*	NN055349	T	OL627827	NA	NA	NA	NA
*Xylolentia reniformis*	GZCC 18-0048		MK547646	MK547648	NA	NA	NA
*Xylolentia simplex*	R. Kirschner 5524		OQ146961	OQ146974	NA	NA	LC745948
*Xylolentia simplex*	R. Kirschner 5618		OQ146962	NA	NA	NA	NA
*Xylolentia subhyalina*	KUNCC 24-17948	T	PQ168259	PQ152651	PQ218171	PV443845	PV443853
*Xylolentia subhyalina*	KUNCC 10481		PQ168260	PQ152652	NA	NA	NA
***Xylolentia yibinensis***	**GZCC 26-0006**		**PX927611**	**PZ000585**	**PZ071917**	**NA**	**NA**
*Xylolentia yibinensis*	GMBC5367	T	PV951777	PV951771	PV961435	PX392374	PX373392
*Xylolentia yibinensis*	GMBC5368		PV951778	PV951772	PV961436	PX392375	PX373393

Maximum likelihood (ML) and Bayesian inference (BI) analyses were conducted on both single-locus and concatenated datasets. The ML analysis was performed using RAxML-HPC v.8 on the CIPRES Science Gateway under the GTR+GAMMA model, with 1,000 bootstrap replicates. Bootstrap support (ML-BS) values ≥ 75% are indicated at the corresponding nodes.

Bayesian inference was conducted in MrBayes 3.2.6 (Ronquist et al. 2012) using a Markov chain Monte Carlo (MCMC) algorithm. For each gene partition, the GTR+I+G substitution model was selected based on the AIC in MrModeltest 2.2 (Nylander 2004). Two parallel runs of six simultaneous Markov chains were performed for 10,000,000 generations. Trees were sampled every 1,000^th^ generation. The burn-in phase was set at 25%, and the remaining trees were used for calculating posterior probabilities (PP). PP values equal to or greater than 0.95 are marked near each node.

Phylogenetic trees were visualized using FigTree v1.4.4 (http://tree.bio.ed.ac.uk/software/figtree; accessed on 30 March 2026) and subsequently annotated with TVBOT (Xie et al. 2023). Final figures were prepared and exported in JPG format using Adobe Photoshop CS6 Extended 10.0 (Adobe Systems, San Jose, CA, USA).

## Results

### Phylogenetic analyses

To elucidate the evolutionary relationships among the four species and their close relatives, two distinct phylogenetic analyses were conducted.

**Figure 1.**
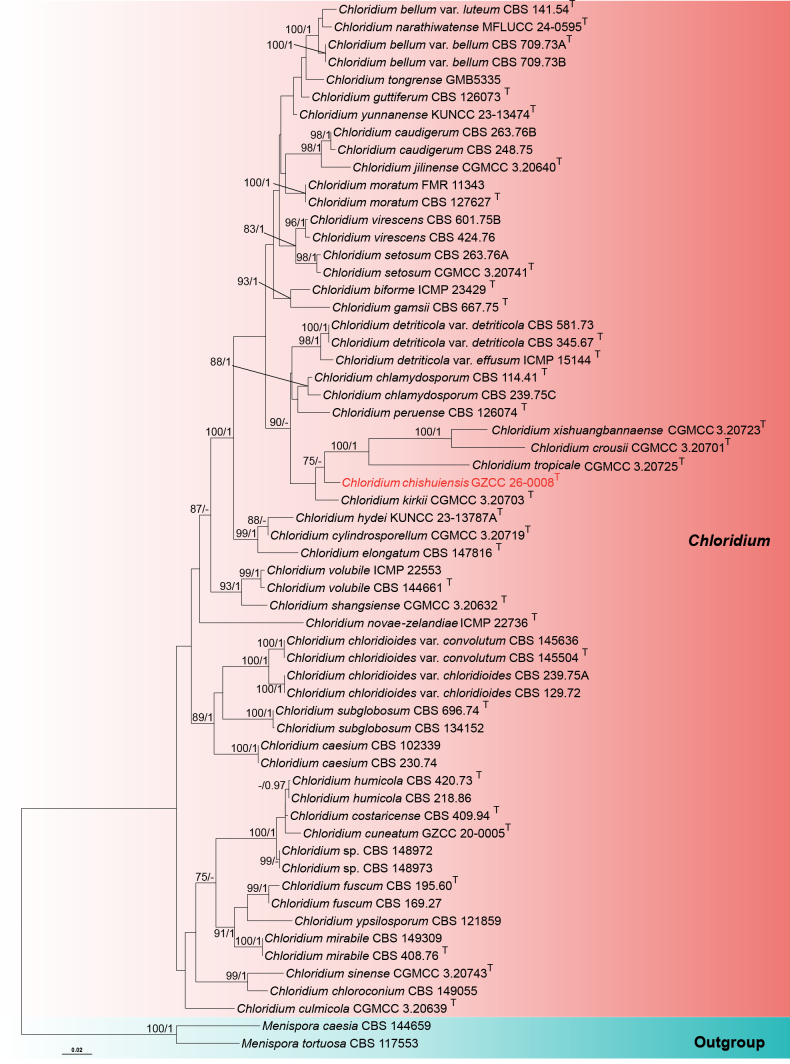
ML tree based on the combined LSU–ITS–*tef*1-α sequences. Bootstrap support values for ML greater than 75% and PP greater than 0.95 are given near nodes as ML-BS/PP. The tree is rooted with *Menispora caesia* (CBS 144659) and *M. tortuosa* (CBS 117553). The superscript “T” denotes ex-type strains. The new taxon is indicated in red.

Analysis 1: The phylogenetic analysis of the combined LSU–ITS–*tef*1-α sequence dataset, showing the phylogenetic relationships of *Chloridium* species, is presented in Fig. 1. The dataset comprises 60 strains, with *Myrmecridium schulzeri* (CBS 100.54) and *M. sorbicola* (CBS 143433) as the outgroup. The aligned dataset consisted of 2,260 total characters (LSU = 817 bp, ITS = 503 bp, *tef*1-α = 940 bp), including gaps. Fig. 1 displays the best-scoring ML tree (–ln = 10430.156610). Multigene phylogenetic analyses demonstrated that the newly generated strain, GZCC 26-0008, represents a distinct lineage and is closely related to *C. crousii* (CGMCC 3.20701), *C. kirkii* (CGMCC 3.20703), *C. tropicale* (CGMCC 3.20725), and *C. xishuangbannaense* (CGMCC 3.20723), with 75% ML-BS support but low PP support. However, the new strain GZCC 26-0008 forms a stable lineage basal to *C. crousii* (CGMCC 3.20701), *C. tropicale* (CGMCC 3.20725), and *C. xishuangbannaense* (CGMCC 3.20723) in both ML and BI analyses, as well as in the ITS phylogenetic tree (Suppl. material 1: fig. S1). Hence, the new species, *Chloridium chishuiensis*, is introduced herein. Unfortunately, many *Chloridium* species were not well resolved in the present phylogenetic analyses, including *C. bellum* var. *luteum* (CBS 141.54), *C. costaricense* (CBS 409.94), *C. culmicola* (CGMCC 3.20639), *C. cuneatum* (GZCC 20-0005), *C. guttiferum* (CBS 126073), *C. humicola* (CBS 420.73 and CBS 218.86), *C. kirkii* (CGMCC 3.20703), *C. narathiwatense* (MFUCC 24-0595), *C. novae-zelandiae* (ICMP 22736), *C. peruense* (CBS 126074), *C. tongrense* (GMB5335), *C. ypsilosporum* (CBS 121859), and *C. yunnanense* (KUNCC 23-13474), concurring with Liu et al. (2025). However, these species formed well-separated branches from related taxa, except for *C. humicola* (CBS 420.73 and CBS 218.86), *C. costaricense* (CBS 409.94), and *C. cuneatum* (GZCC 20-0005), which appear to be conspecific and require further studies.

**Figure 2.**
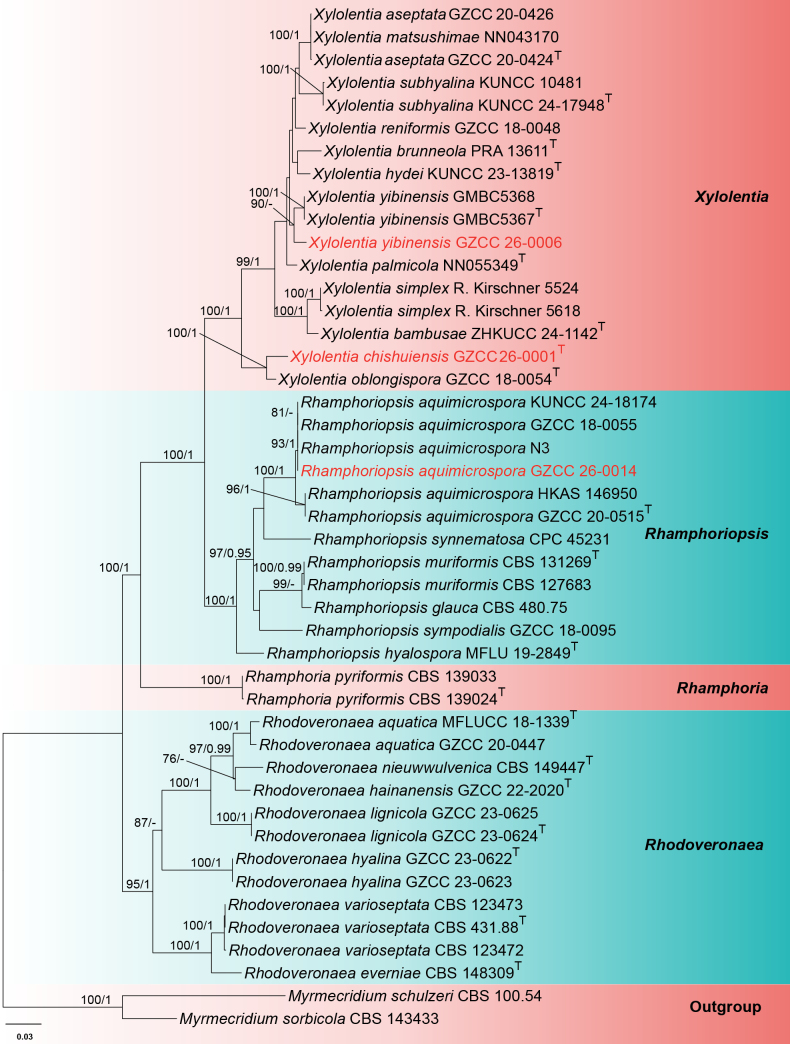
ML tree based on the combined LSU–ITS–SSU–*rpb*2–*tef*1-α sequences. Bootstrap support values for ML greater than 75% and PP greater than 0.95 are given near nodes as ML-BS/PP. The tree is rooted with *Myrmecridium schulzeri* (CBS 100.54) and *M. sorbicola* (CBS 143433). The superscript “T” denotes ex-type strains. The new taxa are indicated in red.

Analysis 2: The phylogenetic analysis of *Rhamphoriaceae* is illustrated in Fig. 2, based on a combined alignment of LSU, ITS, SSU, *rpb*2, and *tef*1-α sequences. The dataset comprised 45 representative taxa in *Rhamphoriaceae*, with *Myrmecridium schulzeri* (CBS 100.54) and *M. sorbicola* (CBS 143433) as the outgroup taxa. The alignment consisted of 4,106 characters (LSU = 813 bp, ITS = 539 bp, SSU = 925 bp, *rpb*2 = 978 bp, and *tef*1-α = 851 bp), including gaps. The ML analysis yielded the best-scoring tree (–ln = 17669.514352) and is selected to represent the relationships among taxa. In these analyses, four genera, *Xylolentia* (ML-BS = 100%, PP = 1), *Rhamphoriopsis* (ML-BS = 100%, PP = 1), *Rhamphoria* (ML-BS = 100%, PP = 1), and *Rhodoveronaea* (ML-BS = 95%, PP = 1), formed well-supported monophyletic subclades, respectively. Within the *Xylolentia* subclade, the new species *X. chishuiensis* (GZCC 26-0001) constitutes a distinct branch and is sister to *X. oblongispora* (GZCC 18-0054), with 100% ML-BS and 1 PP support. Strain GZCC 26-0006 groups with *X. yibinensis* (GMBC5367 and GMBC5368), with 90% ML-BS support but low PP support in BI analysis. Although GZCC 26-0006 exhibits a different branch length compared to GMBC5367 and GMBC5368, these three strains are regarded as conspecific due to low genetic differences (detailed in the notes for *Xylolentia yibinensis*). In the *Rhamphoriopsis* subclade, the new strain GZCC 26-0014 clusters with *R. aquimicrospora* (strains N3, GZCC 18-0055, and KUNCC 24-18174), with 93% ML-BS and 1 PP support, sharing identical branch lengths. However, it is distinct from the type strain (GZCC 20-0515) of *R. aquimicrospora* and strain HKAS 146950, with 100% ML-BS and 1 PP support values. Notably, *X. brunneola* (PRA 13611), *X. hydei* (KUNCC 23-13819), *X. palmicola* (NN055349), *Xylolentia reniformis* (GZCC 18-0048), *Rhamphoriopsis synnematosa* (CPC 45231), and *R. sympodialis* (GZCC 18-0095) were not well resolved in the present study. Furthermore, *Xylolentia aseptata* (GZCC 20-0424 and GZCC 20-0426) is shown to be conspecific with *X. matsushimae* (NN 043170). However, *X. matsushimae* (NN 043170) is not the ex-type strain of *X. matsushimae*. Similarly, *Rhamphoriopsis glauca* (CBS 480.75) clusters with *R. muriformis* (CBS 131269 and CBS 127683), with 99% ML-BS support, and the conspecific status of these two species is also questionable.

### Taxonomy

***Chaetosphaeriaceae* Réblová, M.E. Barr & Samuels, Sydowia 51(1): 56 (1999)**

***Chloridium* Link, Mag. Gesell. naturf. Freunde, Berlin 3(1–2): 13 (1809)**

#### Chloridium chishuiensis

Taxon classificationFungiChaetosphaerialesChaetosphaeriaceae

H Zhang & Y.R. Sun sp. nov.

B67C21F3-45B4-54B0-B66B-CB63D19F7A38

Fungal Names: FN 573528

Fig. 3

##### Etymology.

The epithet refers to the Chishui River Basin, the location where the taxon was collected.

##### Holotype.

GZAAS 26-0005.

##### Description.

***Saprobic*** on submerged decaying wood in the Chishui River. **Asexual morph**: Hyphomycetous. ***Colonies*** effuse, brown, hairy, with white glistening conidial masses at the apex. ***Setae*** absent. ***Conidiophores*** 88–155 × 2.5–5 µm (x̄ = 118 × 3.5 µm, *n* = 15), solitary or 2–4 in group, cylindrical, erect, unbranched, straight or slightly flexuous, 3–6-septate, dark brown at the base, becoming paler towards the apex, tapering gradually towards the apex, smooth, arrowing to 2 µm wide at the upper part below the collarettes. ***Conidiogenous cells*** 23–47 × 1.5–2.5 µm (x̄ = 31.5 × 2 µm, *n* = 15), phialidic, enteroblastic, integrated, terminal, cylindrical to subcylindrical, pale brown to subhyaline, paler towards the tip, slightly narrower below the collarette; collarettes short, flaring, subhyaline, smooth-walled, with multi conidiogenous loci within the collarettes. ***Conidia*** 3–4.5 × 1.5–2.5 µm (x̄ = 3.7 × 2 µm, *n* = 25) singly, within the collarette, aggregated in wet spore mass, hyaline, oblong to ellipsoidal, straight, aseptate, guttulate. **Sexual morph**: Undetermined.

**Figure 3.**
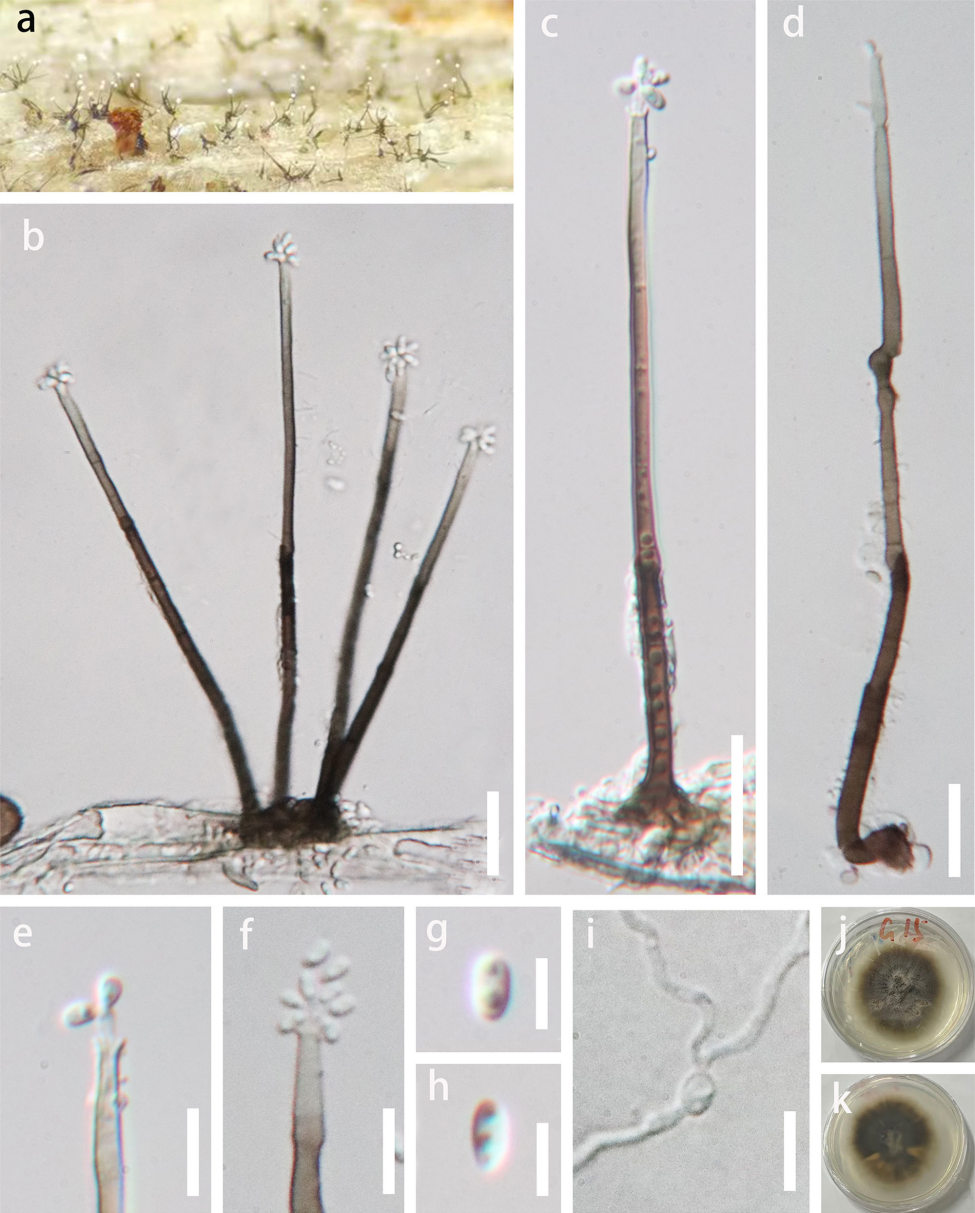
*Chloridium chishuiensis* (GZAAS 26-0005, holotype). **a**. Colonies on natural substrate; **b–d**. Conidiophores with conidia; **e, f**. Conidiogenous cells with conidia; **g, h**. Conidia; **i**. Germinated conidium; **j, k**. Colonies on PDA (reverse view and top view). Scale bars: 20 μm (**b–d**), 10 μm (**e, f, i**), 5 μm (**g, h**).

##### Culture characteristics.

Conidia germinated on PDA within 12 hours at 25 °C. Germ tubes were produced from both ends of conidia. Colony reaching 20 mm in diameter after 4 weeks at room temperature on PDA media, circular, with entire, even margins. Mycelia are dry, flat, and appressed to the medium, with a finely felty texture in the central area, no raised structures, aerial mycelium tufts, or exudates. Obverse: dark brown to nearly black in the center, fading to light brown towards the margin, without concentric rings or radial striations. Reverse: uniformly dark blackish-brown, non-diffusible pigment, no discoloration of the surrounding PDA medium.

##### Material examined.

China • Guizhou Province, Zunyi City, Renhuai City, Maotai Town, on submerged decaying wood in the Chishui River, 5 October 2025, H Zhang, Z15 (GZAAS 26-0005, holotype, ex-type culture GZCC 26-0008).

##### Notes.

The BLASTn searches of the LSU sequence of *Chloridium chishuiensis* resulted in 99.63%, 99.39%, and 99.26% matches with *C. kirkii* (NN043888), *C. chlamydosporum* (CBS 423.76), and *C. peruense* (CBS 126074), respectively. For the ITS region, the closest matches were predominantly with uncultured environmental sequences, with the highest similarity to an uncultured fungus (GenBank accession no. HM030609) at 97.2% similarity. Among named species, the closest match was with *C. chlamydosporum* (GenBank accession no. MH858992) at 96.3% similarity. The multi-gene phylogeny (Fig. 2) showed that *C. chishuiensis* is related to *C. crousii*, *C. kirkii*, *C. tropicale*, and *C. xishuangbannaense*. Pairwise comparisons of ITS sequences (gaps excluded) showed that *C. chishuiensis* differs from *C. crousii*, *C. kirkii*, *C. tropicale*, and *C. xishuangbannaense* by 9.3% (45/482 bp), 2.9% (14/482 bp), 7.5% (36/482 bp), and 8.9% (43/482 bp), respectively. The ITS sequence divergence between *C. chishuiensis* and related species is well above the 1.5% threshold proposed by Jeewon and Hyde (2016) for species recognition.

Morphologically, *Chloridium chishuiensis* differs from *C. crousii*, *C. tropicale*, and *C. xishuangbannaense* in producing multiple conidiogenous loci within the collarettes, whereas the latter possess a single sporulating locus within the collarettes (Wu and Diao 2022). It resembles *C. kirkii* but can be distinguished by its longer conidiophores (88–155 × 2.5–5 µm vs. 22–65 × 2–2.5 µm) and guttulate conidia (Wu and Diao 2022). In addition, chlamydospores were not observed in this species. Consequently, *C. chishuiensis* is proposed here as a new species based on both molecular and morphological evidence.

### *Rhamphoriaceae* Réblová, Mycologia 110(4): 754 (2018)

***Xylolentia* Réblová, in Réblová & Štěpánek, Mycologia 110(4): 759 (2018)**

#### Xylolentia chishuiensis

Taxon classificationFungiRhamphorialesRhamphoriaceae

Y.R. Sun & H Zhang sp. nov.

6AF86562-CC45-5F86-8C72-950E3CD07D2D

Fungal Names: FN 573529

Fig. 4

##### Etymology.

The epithet refers to the Chishui River Basin, the location where the taxon was collected.

##### Holotype.

KUN-HKAS 154381.

##### Description.

***Saprobic*** on submerged bamboo culms in the Chishui River. **Asexual morph**: Hyphomycetous. ***Colonies*** on natural substrate effuse, white, hairy, visible as solitary, brown conidiophores with white conidial masses at the apex. ***Mycelium*** immersed, composed of septate, branched, hyaline hyphae. ***Conidiophores*** 80–110 μm long, 3–4 μm wide, macronematous, mononematous, solitary, erect, straight or curved, cylindrical, unbranched, 6–9-septate, brown, becoming pale brown at the apex, smooth-walled, tapering towards the apex. ***Conidiogenous cells*** 20–38 × 2.5–3.5 μm (x̄ = 28 × 3 μm, *n* = 15), polyblastic, integrated, terminal, determinate, cylindrical to cylindric-lageniform, pale brown near the base, subhyaline to hyaline towards the apex. ***Conidia*** 2.5–4 × 1.5–2.5 μm (x̄ = 3 × 1.8 μm, *n* = 50), acrogenous, aggregated in slimy masses, ellipsoidal to subglobose, aseptate, hyaline, smooth-, thin-walled. **Sexual morph**: Undetermined.

**Figure 4.**
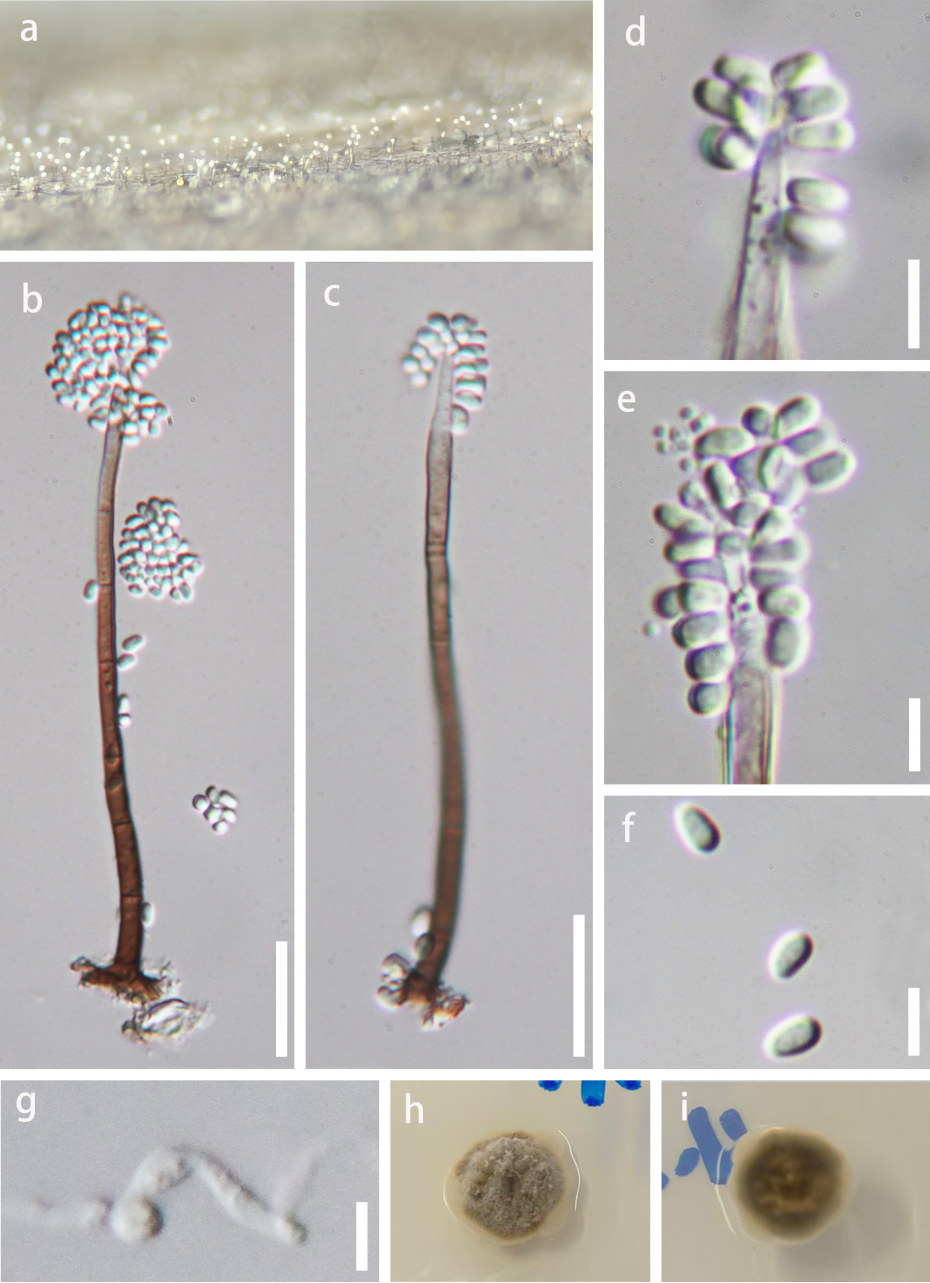
*Xylolentia chishuiensis* (KUN-HKAS 154381, holotype). **a**. Colonies on natural substrate; **b, c**. Conidiophores with conidia; **d, e**. Conidiogenous cells with conidia; **f**. Conidia; **g**. Germinated conidium; **h, i**. Colonies on PDA (reverse view and top view). Scale bars: 20 μm (**b, c**), 5 μm (**d–g**).

##### Culture characteristics.

Conidia germinated on PDA within 12 hours at 25 °C. Germ tubes were produced from both ends. Colonies on PDA reaching 20 mm in diam after 4 weeks at room temperature, circular, entire, moderate mycelial density, raised-convex. Surface dry, velvety-floccose, matte; obverse centrally dark brown, peripherally pale brown; reverse uniformly dark brown, no diffusible pigment. Mycelia woolly, centrally compact, marginally loose-floccose.

##### Material examined.

China • Guizhou Province, Zunyi City, Renhuai City, Maotai Town, on submerged bamboo culms in the Chishui River, 4 October 2025, H. Zhang, Z112 (KUN-HKAS 154381, holotype, ex-type culture GZCC 26-0001; ibid., GZAAS 26-0004, isotype).

##### Notes.

BLAST searches of the newly generated sequences against the NCBI Nucleotide Database confirmed the genetic distinctiveness of the taxon, with sequence identities of 95.46% (ITS), 99.50% (LSU), and 99.89% (SSU) relative to the closest sequences from the same genus. *Xylolentia chishuiensis* is phylogenetically sister to *X. oblongispora* (GZCC 18-0054) with high support (ML-BS = 100%, PP = 1) (Fig. 2). Morphologically, *X. chishuiensis* can be distinguished from *X. oblongispora* by its shorter and narrower conidiophores (80–110 × 3–4 μm vs. 91–219 × 3.5–8.5 μm) and its smaller, ellipsoidal to subglobose conidia (2.5–4 × 1.5–2.5 μm vs. 3.6–5.3 × 2.2–3.2 μm) (Lin et al. 2025). In contrast, *X. oblongispora* produces oblong conidia with obtuse ends and guttules (Lin et al. 2025). Additionally, the two species exhibit a difference of 17/502 base pairs (3.4%, without gap) in the ITS region and 20/850 base pairs (2.4%, without gap) in the *tef*1-α region. Based on this combined molecular and morphological evidence, *Xylolentia chishuiensis* is described as a new species.

#### Xylolentia yibinensis

Taxon classificationFungiRhamphorialesRhamphoriaceae

L.L. Liu, K. Habib & Q.R. Li, Mycosphere 16(1): 4045 (2025)

492C008E-6C77-5303-892E-06962CB1C233

Fig. 5

##### Description.

***Saprobic*** on submerged decaying wood in the Chishui River. **Asexual morph**: Hyphomycetous. ***Colonies*** on natural substrate effuse, hairy, scattered, or aggregated, with white glistening conidial masses at the apex. ***Mycelium*** immersed, composed of septate, branched, hyaline hyphae. ***Conidiophores*** 60–80 µm long, 2.5–4 µm wide, macronematous, mononematous, erect, straight or slightly curved, cylindrical, unbranched, 4–8-septate, brown, becoming pale brown to subhyaline at the apex, tapering towards the apex, smooth-walled. ***Conidiogenous cells*** 33–41 × 2.5–3 µm (x̄ = 35 × 2.7 µm, *n* = 15), polyblastic, integrated, terminal, determinate, cylindrical, pale brown to hyaline, smooth-walled. ***Conidia*** 2.5–4 × 1.5–2.5 μm (x̄ = 3.5 × 2 μm, *n* = 30), acrogenous, aggregated in slimy masses, ellipsoidal, subglobose, hyaline, aseptate, guttulate, smooth-walled. **Sexual morph**: Undetermined.

**Figure 5.**
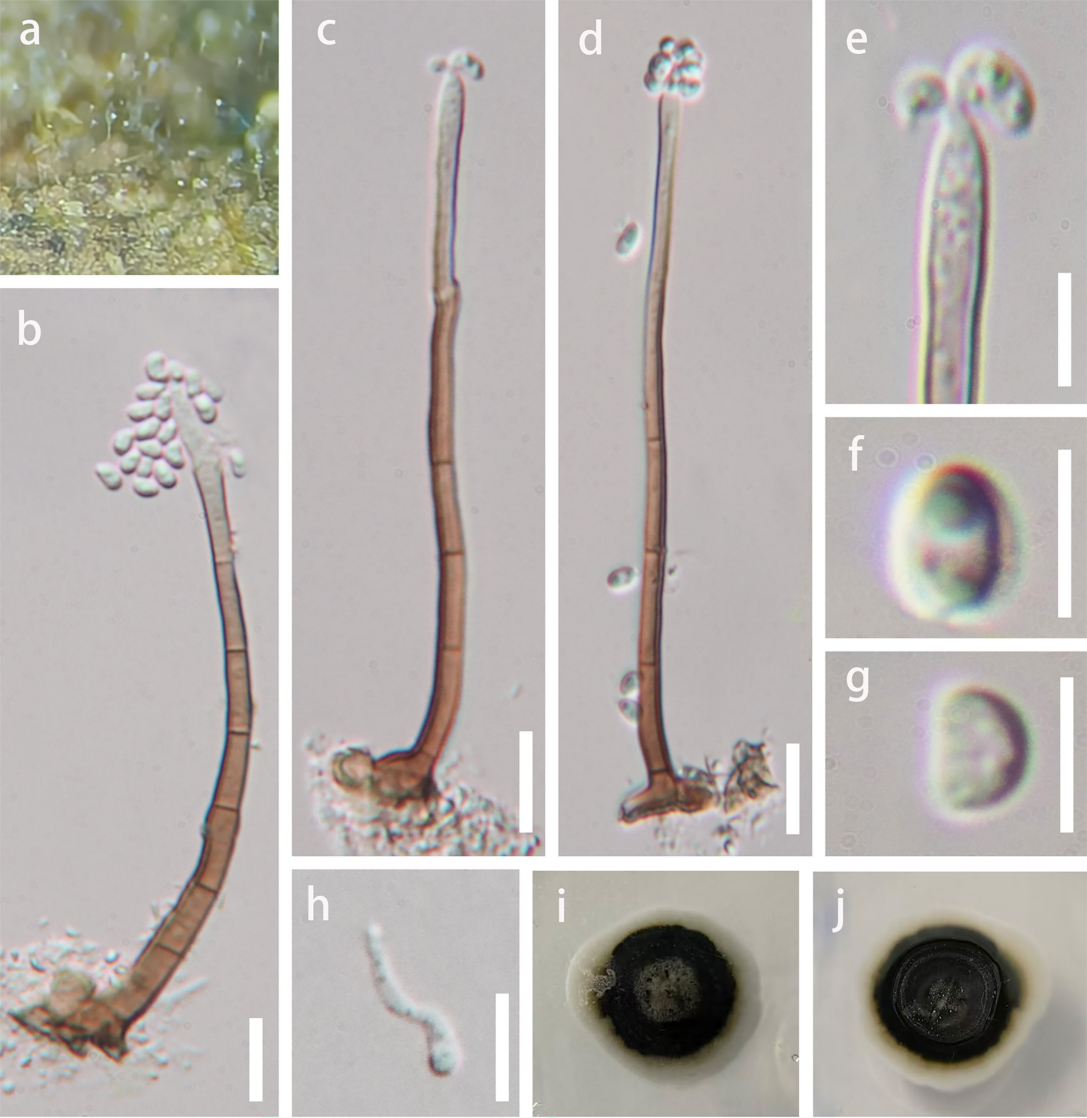
*Xylolentia yibinensis* (KUN-HKAS 154382). **a**. Colonies on natural substrate; **b–d**. Conidiophores with conidia; **e**. Conidiogenous cells with conidia; **f, g**. Conidia; **h**. Germinated conidium; **i, j**. Colonies on PDA (reverse view and top view). Scale bars: 10 μm (**b–d, h**); 5 μm (**e–g**).

##### Culture characteristics.

Conidia germinated on PDA within 12 hours at 25 °C. Germ tubes were produced from one end of the conidia. Colony reaching 15–20 mm in diameter after 4 weeks at room temperature on PDA media, slow-growing, circular, entire, density, raised. Surface dry, felty, matte, zonate: centrally black, dark olive-brown intermediate, gray to white at margin; reverse uniformly dark brown, no diffusible pigment. Mycelia compact-felty centrally, appressed-loose at margin, exudate absent.

##### Material examined.

China • Guizhou Province, Zunyi City, Renhuai City, Maotai Town, on submerged decaying wood in the Chishui River, 5 October 2025, H Zhang, Z211 (KUN-HKAS 154382 = GZAAS 26-0003, living culture GZCC 26-0006).

##### Notes.

*Xylolentia yibinensis* was recently described by Liu et al. (2025) from a freshwater habitat in Sichuan Province, China. The isolate, also collected from a freshwater environment in Guizhou, China, groups with *X. yibinensis* in the phylogenetic analysis (Fig. 2). Morphologically, the specimen resembles *X. yibinensis* in the shape of conidiophores, conidiogenous cells, and conidia, although it possesses longer conidiogenous cells (33–41 µm in the isolate vs. 13.5–25.8 μm in the type). Furthermore, a comparison of DNA sequence data shows that the strain is identical to the ex-type strain of *X. yibinensis* in the LSU and SSU regions but differs by 7/504 base pairs in the ITS region (1.39%, without gaps) and 10/879 base pairs in the *tef*1-α region (1.14%, without gaps). Based on the combination of morphological and molecular evidence, the new collection is identified as *Xylolentia yibinensis*. The species is reported from Guizhou Province, China, for the first time.

### *Rhamphoriopsis* Réblová & Gardiennet, Mycologia 110(4): 754 (2018)

#### Rhamphoriopsis aquimicrospora

Taxon classificationFungiRhamphorialesRhamphoriaceae

J. Yang, Jian K. Liu & K.D. Hyde, Fungal Diversity 119: 168 (2023)

0DC7896E-CAE9-5C4F-9AAA-3754E7CD6BCC

Fig. 6

##### Description.

***Saprobic*** on bamboo culms submerged in the Chishui River. **Asexual morph**: Hyphomycetous. ***Colonies*** effuse, scattered or in small groups, hairy, visible as solitary, brown conidiophores with white conidial masses at the apex. ***Mycelium*** mostly immersed, composed of septate, smooth-walled, pale brown to hyaline hyphae. ***Conidiophores*** 105–150 µm long, 7–11 µm wide at middle part, up to 30 µm wide at the base, macronematous, synnematous, erect, septate, cylindrical, reddish brown, paler towards the apex, with terminal of conidiophores splaying out as a flared head. ***Conidiogenous cells*** 1–2 µm wide, polyblastic, integrated, terminal, pale brown near the base, becoming hyaline towards the apex, smooth-walled. ***Conidia*** 2–3 × 1–2 µm (x̄ = 2.5 × 1.5 µm, *n* = 30), ellipsoidal, aseptate, smooth-walled, hyaline. **Sexual morph**: Undetermined.

**Figure 6.**
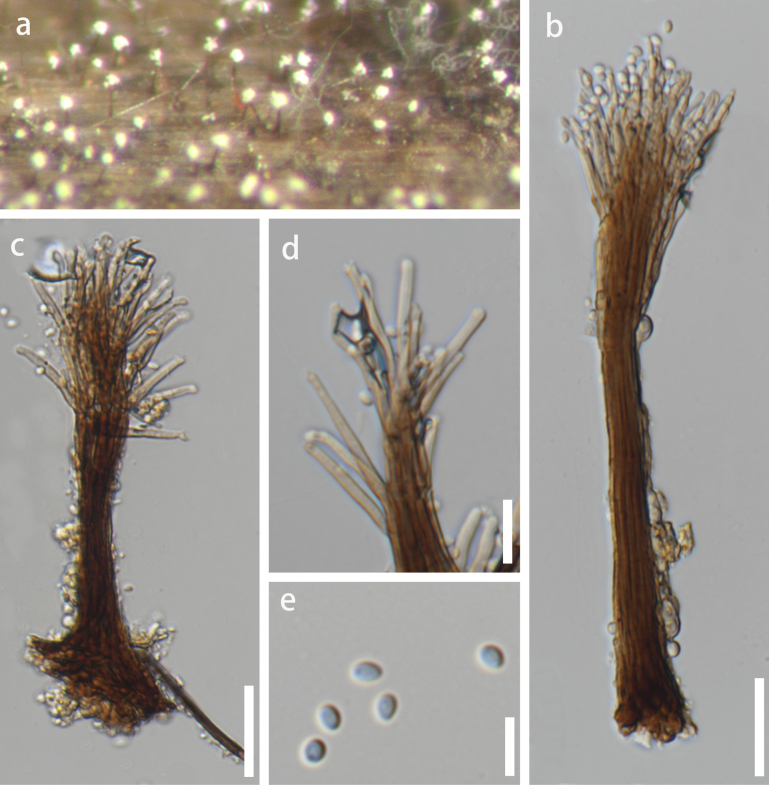
*Rhamphoriopsis aquimicrospora* (KUN-HKAS 154383). **a**. Colonies on natural substrate; **b, c**. Conidiophores; **d**. Conidiogenous cells; **e**. Conidia. Scale bars: 20 μm (**b, c**); 10 μm (**d**); 5 μm (**e**).

##### Culture characteristics.

Conidia germinated on PDA within 12 hours at 25 °C. Germ tubes were produced from both ends. Colony reaching 20–25 mm in diameter after 6 weeks at room temperature on PDA media, circular, raised, waxy-mucoid, with sparse, finely, floccose, light yellow aerial mycelium on the surface; in reverse yellow with entire margin.

##### Material examined.

China • Guizhou Province, Zunyi City, Renhuai City, Maotai Town, on bamboo culms submerged in the Chishui River, 5 July 2025, H Zhang, Z279 (KUN-HKAS 154383, living culture GZCC 26-0014)

##### Notes.

*Rhamphoriopsis aquimicrospora* was first documented from submerged decaying twigs in a waterfall (Guizhou Province, China) by Yang et al. (2023). Later, Lin et al. (2025) reported its first occurrence in terrestrial habitats in Guizhou Province. In the present study, a fungal collection obtained from bamboo is examined. BLASTn analyses revealed that the ITS sequence shared 98.8% similarity with the ex-type strain of *R. aquimicrospora* and 98.7%–100% identity with other conspecific accessions, whereas the LSU sequence showed 99.6% similarity to the ex-type and 99.8%–100% identity among conspecific strains. Morphologically, it aligns with the description of *R. aquimicrospora*, although the synnemata in the specimen are shorter (105–150 µm) compared to the holotype (135–178 µm; Yang et al. 2023). Phylogenetic analysis placed the strain within a clade comprising other strains of *R. aquimicrospora* (Fig. 2). Sequence comparison of the LSU, ITS, and SSU regions between the strain and the ex-type strain (GZCC 20-0515) revealed 1/779 bp (0.13%), 3/517 bp (0.58%), and 0/907 bp differences, respectively. Based on morphological and molecular evidence, the collection is identified as *Rhamphoriopsis aquimicrospora*, which represents a new host record for this species.

## Discussion

In this study, two new species in *Chloridium* and *Xylolentia*, namely *Chloridium chishuiensis* and *Xylolentia chishuiensis*, a new geographical record in *Xylolentia*, and a new host record of *Rhamphoriopsis* are introduced, contributing to the freshwater fungal diversity of the Chishui River Basin.

Within the phylogenetic tree of *Chloridium*, the subclade comprising *C. cuneatum* (GZCC 20-0005), *C. costaricense* (CBS 409.94), and *C. humicola* (CBS 420.73 and CBS 218.86) is not well resolved (Fig. 2). In the ITS single-gene analyses (Suppl. material 1: fig. S1), *Chloridium* sp. (CBS 148972 and CBS 148973), *C. costaricense* (CBS 409.94), and *C. humicola* (CBS 420.73 and CBS 218.86) are not clearly resolved. Conversely, the *tef*1-α phylogeny (Suppl. material 1: fig. S2) differentiates these taxa into distinct lineages, despite the short branch lengths observed between *C. costaricense* and *C. humicola*. Consequently, *tef*1-α appears to be a superior marker for species delimitation within *Chloridium*. Nevertheless, further investigation is needed to clarify the conspecific status of these isolates.

A critical challenge in fungal systematics is the delineation of closely related species. In the present study, *Xylolentia yibinensis* exhibits high ITS sequence similarity with *X. palmicola*, differing by only five base pairs. However, despite this low genetic divergence in the ITS region, multi-locus phylogenetic analysis revealed that these taxa do not cluster together, and they are morphologically distinct (Wu and Diao 2022; Liu et al. 2025). This discrepancy may be partly attributed to data limitations: *Xylolentia palmicola* is represented only by ITS sequence data, with no other genes (e.g., LSU and *tef*1-α) available to provide greater phylogenetic resolution. A definitive resolution of these species’ boundaries will ultimately require additional collections and multi-locus data.

Notably, two isolates of *X. aseptata* formed a well-supported monophyletic subclade with *X. matsushimae* in the present study. Wu and Diao (2022) transferred *Chloridium reniforme* to *Xylolentia* as *X. matsushimae* based on phylogenetic analyses. Subsequently, Yang et al. (2023) described *Xylolentia aseptata* as a new species isolated from Guizhou Province, China. Only ITS sequence data are currently available for *X. matsushimae* (non-type strain), and pairwise comparison of the ITS sequences revealed only a single gap difference across a 523-bp alignment. Morphologically, *X. matsushimae* and *X. aseptata* exhibit highly similar morphology. Although the conidiophores of *X. matsushimae* are comparatively shorter (60–160 μm) than those of *X. aseptata* (95–215 μm), the two taxa display overlapping conidial dimensions (3–4 × 2–2.5 μm vs. 3–5.5 × 1.8–3 μm). Despite the minimal nucleotide divergence and the overlapping morphological features, *X. matsushimae* is not represented by the type. Hence, confirming the conspecific status and synonymization of *X. aseptata* under *X. matsushimae* is required for further resolution.

## Supplementary Material

XML Treatment for Chloridium chishuiensis

XML Treatment for Xylolentia chishuiensis

XML Treatment for Xylolentia yibinensis

XML Treatment for Rhamphoriopsis aquimicrospora

## Additional information

### Artificial Intelligence (AI) use

The authors accept full responsibility for the content of the manuscript, including the disclosure of any use of AI.

No AI tools were used in the preparation of this manuscript.

### Author ORCIDs

H. Zhang https://orcid.org/0000-0003-1894-8169

Ya-Ru Sun https://orcid.org/0000-0001-5549-1028

Y. Luo https://orcid.org/0009-0008-2873-6972

J. Zhang https://orcid.org/0009-0006-7210-2630

T. Fan https://orcid.org/0009-0007-4175-7973

X. Liu https://orcid.org/0000-0003-1153-5898

N.-G. Liu https://orcid.org/0000-0002-9169-2350

Y. Mu https://orcid.org/0000-0002-0697-6229

### Data availability

All of the data that support the findings of this study are available in the main text or Supplementary Information.

## Supplementary materials

Supplementary material 1 Supplementary figures Data type docx Explanation note **fig. S1**. ML tree (for *Chloridium*) based on the ITS sequences. The tree is rooted with *Menispora caesia* (CBS 144659) and *M. tortuosa* (CBS 117553). **fig. S2**. ML tree (for *Chloridium*) based on the *tef*1-α sequences. The tree is rooted with *Menispora caesia* (CBS 144659) and *M. tortuosa* (CBS 117553). https://binary.pensoft.net/file/1613531This dataset is made available under the Open Database License (http://opendatacommons.org/licenses/odbl/1.0/). The Open Database License (ODbL) is a license agreement intended to allow users to freely share, modify, and use this Dataset while maintaining this same freedom for others, provided that the original source and author(s) are credited. Hong Zhang, Ya-Ru Sun, Yongjian Luo, Jiayi Zhang, Tianyuan Fan, Xudong Liu, Ning-Guo Liu, Yu Mu
