# Supplementary material for: Four Sordariomycetes freshwater hyphomycetes from the Chishui River Basin in Guizhou Province, China
Source: MycoKeys. 2026 Apr 29;131:305–26. doi: 10.3897/mycokeys.131.185643 (PMC13150535; doi:10.3897/mycokeys.131.185643)
Supplement: Supplementary material 1 — Supplementary figures [file mycokeys-131-305-s001.docx]

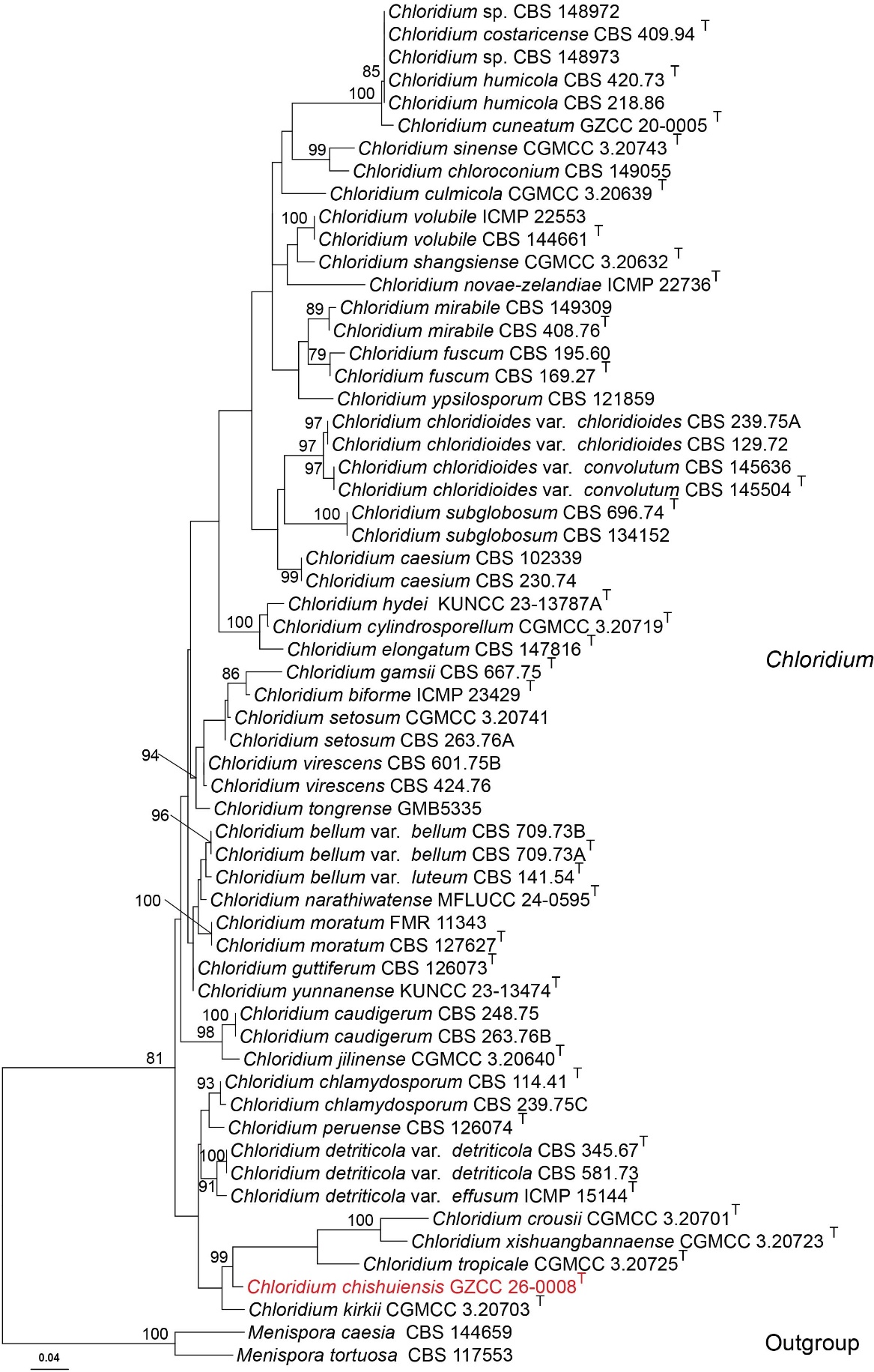


**Figure 1.** ML tree (for *Chloridium*) based on the ITS sequences. The tree is rooted with *Menispora caesia* (CBS 144659) and *M. tortuosa* (CBS 117553).


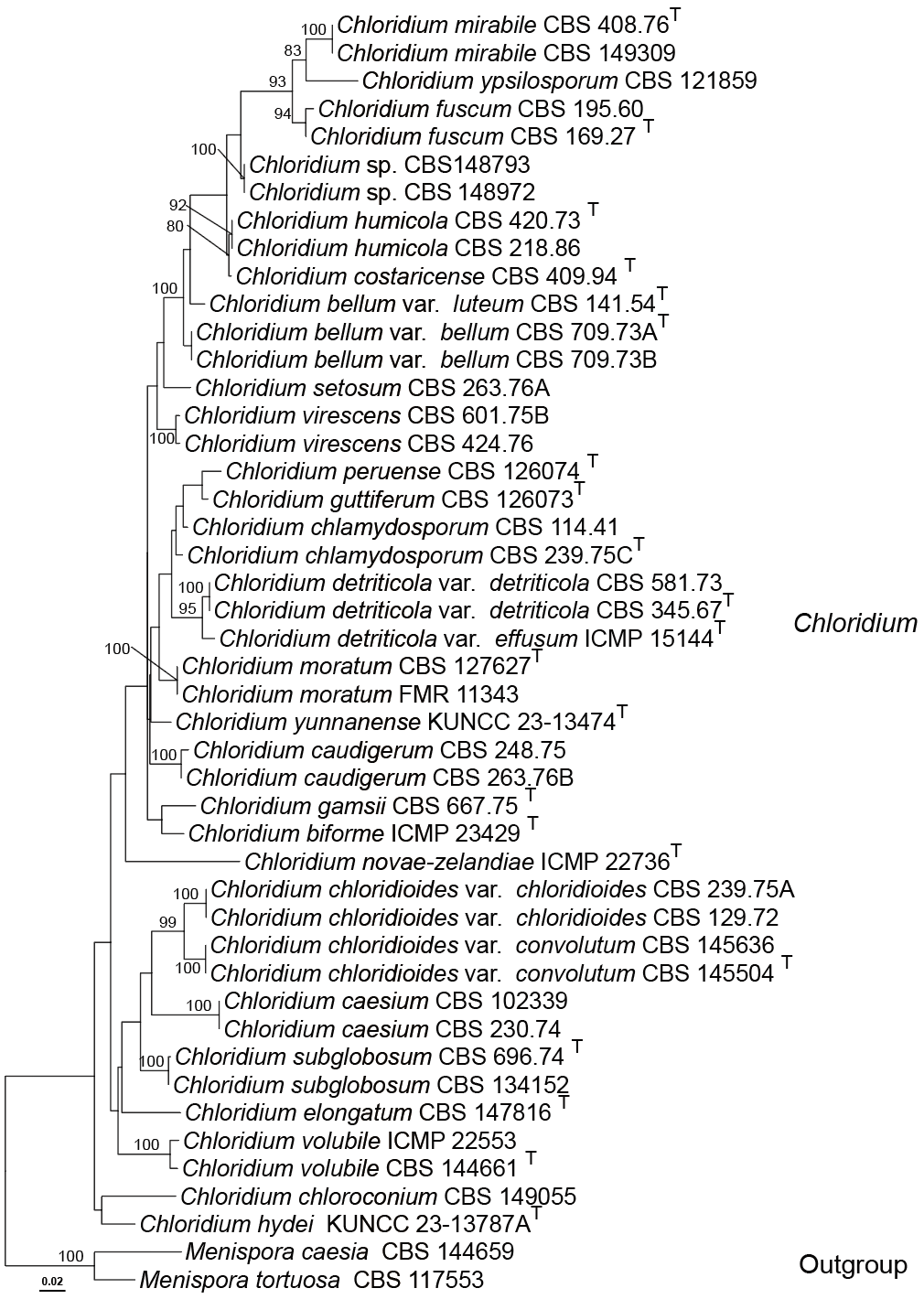


**Figure 2.** ML tree (for *Chloridium*) based on the *tef*1-α sequences. The tree is rooted with *Menispora caesia* (CBS 144659) and *M. tortuosa* (CBS 117553).
